# Spatial–temporal distribution of global production–living–ecological space during the period 2000–2020

**DOI:** 10.1038/s41597-023-02497-1

**Published:** 2023-09-07

**Authors:** Jingying Fu, Qiang Gao, Dong Jiang, Xiang Li, Gang Lin

**Affiliations:** 1 Institute of Geographic Sciences and Natural Resources Research, CAS, Beijing, 100101 China; 2 College of Resources and Environment, University of Chinese Academy of Sciences, Beijing, 100049 China; 3 Key Laboratory of Coupling Processes and Effects of Natural Resource Elements, Ministry of Natural Resources, Beijing, 100055 China; 4 Key Laboratory of Carrying Capacity Assessment for Resource and Environment, Ministry of Natural Resources, Beijing, 100812 China; 5 College of Geoscience and Surveying Engineering, China University of Mining and Technology (Bejing), Beijing, 100083 China

**Keywords:** Geography, Environmental social sciences, Agriculture

## Abstract

Global production-living-ecology space closely corresponds with sustainable development’s economic, social and ecological elements. The dataset of global production–living–ecological space in this paper was generated by combining the global land cover obtained using GlobeLand30 and the population density supplied by NASA’s Socioeconomic Data and Applications Center in 2000, 2010, and 2020. The verification was carried out using the random sampling function of ArcGIS software on the basis of Google Earth sample images. The overall accuracy of the global production–living–ecological space data in 2020 was 83.94% and the Kappa coefficient was 0.81. The overall accuracy of the global production–living–ecological space data in 2010 was 87.00% and the Kappa coefficient was 0.84. The overall accuracy of the spatial data in 2000 was 86.06% and the Kappa coefficient was 0.83. The dataset fills a gap in the global production-living-ecology space database and will be an essential reference for assessing the coordinated development of sustainable development goals.

## Background & Summary

Research on land use multifunctionality is an important academic proposition in land science and geography, and is of important theoretical and practical value for improving land use efficiency and promoting regional sustainable development. Land use function not only includes the service function for meeting the needs of human production and living, but also the ecological function and the self-regulation function—namely, the three categories of land corresponding to production land, living land and ecological land. Land for production refers to land for agricultural, industrial, and commercial activities to obtain products and supply functions, land for living is land for carrying and safeguarding human settlement functions, and land for ecology is land for regulating, maintaining and safeguarding ecological security functions. Among them, ecological land is the foundation, which is the premise of supporting production land and living land to realize their own functions. To ensure the important basic status of ecological land use and to be able to realize intensive production, harmonious life and ecological balance keys in coordinating the man–land relationship and achieving sustainable regional development. However, the current land use classification system places a greater focus on the production and living functions of land, while not sufficiently considering the ecological characteristics. Much land that possesses important ecological value is classified as unused land, which makes the difference between “unused land” and other land types (e.g., “agricultural land” and “construction land”) highly arbitrary^1^. Therefore, it is urgent to build a classification system that focuses on the land use function, emphasizes the role of ecological function in the classification system, thus bringing ecological land into the land use classification system, and coordinating production, life and ecological land space. Recently, some studies have begun to explore the concept and connotation of ecological land use^2–4^, involving regional ecological land classification research^5–7^, and conceptually attempting to construct a classification system for production, living and ecological land use in typical areas^8–10^. However, most of the relevant results have re-merged the original land classification system and propose conceptual frameworks^11–13^. There is a lack of research work that has been clearly implemented in space. The United Nations 2030 Agenda for Sustainable Development puts forward new requirements for the sustainable development of human society. From the perspective of land sustainability, specific indicators under the framework of the global Sustainable Development Goals (SDGs) should be sorted out and classified in detail. Based on specific national conditions and existing relevant studies, the scientific evaluation of land sustainability, which includes three dimensions—intensive production, harmonious life and ecological balance—is an important key issue^14^. Therefore, on a global scale, it is urgent to put forward a set of operable classification systems for production–living–ecological space, and to establish a scientifically reasonable dataset for production–living–ecological space that ensures that the land use system attaches equal importance to production, living and ecological functions. According to the “element–structure–function” theory in system theory, the structure of a system is the basis of the realization of the system’s function. Thus, regarding land use as a system, and land use structure is the basis of the realization of land use function. This study provides a new dataset for global production–living–ecological space in 2000, 2010 and 2020. This dataset is intended to combine global land cover and population density data based on land use type structure from the perspective of multifunctional land use, and to establish a logical connection between and classification system incorporating land use type and land use function. The aim is to construct a global-scale classification and evaluation system for land use, implementing various types of land use in the classification system of “land use and ecological land use” in space, and analyze their spatial pattern characteristics, so as to guide land management on a scientific basis, coordinate the relationship between different types of land use, and systematically support the evaluation of land sustainability under the SDGs framework^15^.

## Methods

### Procedure

**Fig. 1:**
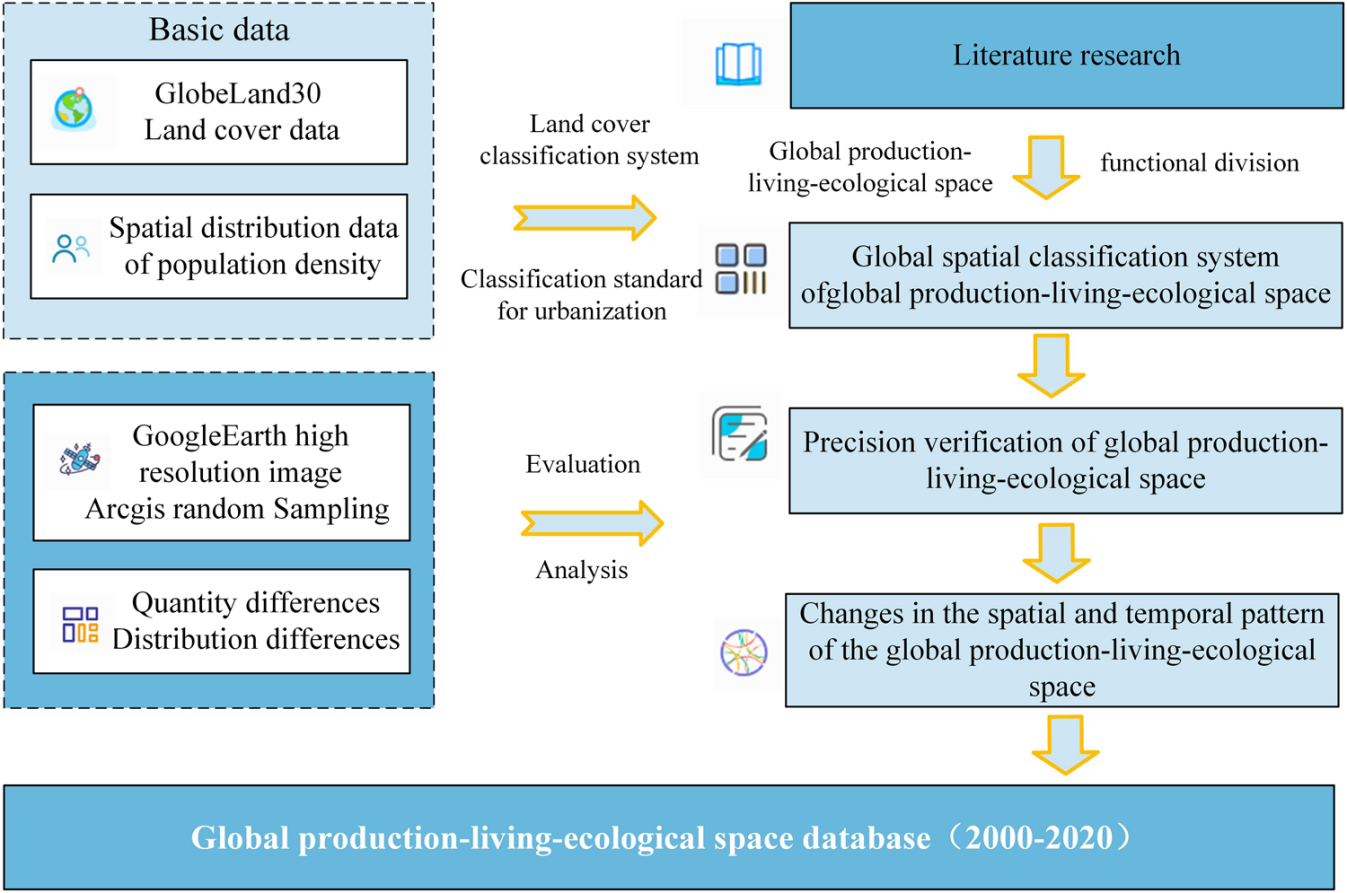
Workflow for creating the dataset of spatial–temporal distribution of global production–living–ecological space during the period 2000–2020.

The global production–living–ecological space database consists of the land cover/land use (LULC) dataset obtained by GlobeLand30 (http://www.globallandcover.com/), the Socioeconomic Data and Applications Center (https://sedac.ciesin.columbia.edu/) population density dataset obtained on the basis of Google Earth high-resolution satellite images, which is generated through reclassification, data merge, random sampling verification and other steps (Fig. 1). The research framework is as follows:

Step 1: The GlobeLand30 land cover data correspond to the classification system of global production–living–ecological space. Woodland and shrub land in the GlobeLand30 land cover data were categorized as woodland ecological space, which included deciduous broad-leaved forest, evergreen broad-leaved forest, deciduous coniferous forest, evergreen coniferous forest, mixed forest, mountain shrub, deciduous shrub, evergreen shrub, etc. Grasslands in GlobeLand30 land cover data were categorized as grassland ecological space, which included grasslands, meadows, savannas, desert grasslands, etc. Wetlands, water bodies, and permanent glacier and snow cover in the GlobeLand30 land cover data were categorized as water ecological space, which included all kinds of swamps, rivers, lakes, reservoirs, pits, and land covered by permanent snow, glaciers and ice caps. Bare land and tundra in the GlobeLand30 land cover data were categorized as other ecological spaces. The cultivated land in GlobeLand30 land cover data was categorized as agricultural production space, including paddy fields, irrigated dry land, rain-fed dry land, vegetable field, pasture growing land, greenhouse land, and land mainly planted with crops, with fruit trees and other economic trees. Artificial land surfaces in the GlobeLand30 land cover data were categorized as industrial production space (the industrial production space, here, is the first step, based on GlobeLand30 land cover data, which includes various residential places such as towns, industrial and mining areas, transportation facilities, etc.). Since it includes a range of residential areas (i.e., living space), industrial production space in the final generated data product needs to be further broken down to determine the range of living space in the industrial production space here.

Step 2: Refer to the classification criteria of the Global Human Settlements Layer (GHSL) Urbanization dataset published by the European Commission. Rural land with a population density of 300–1500 people/grid unit (1 km^2^) was defined as rural land, and land with a population density of more than 1500 people/grid unit (1 km^2^) was defined as urban land^16–18^. According to this standard, using the population density spatial distribution data of NASA’s Socioeconomic Data and Applications Center (SEDAC) and the “reclassification” tool in ArcGIS 10.5, the corresponding types of production–living–ecological space were divided in order to generate the global production–living–ecological spatial data for the years 2000, 2010 and 2020 (Table 1).

**Table 1:** Global production–living–ecological space classification system.

Primary category	Secondary category	Content	Data source
The production space	1- Agricultural production space	Provide direct agricultural products to humans	GlobeLand30: cropland
	2- Industrial production space	Industrial and mining, transportation construction land, etc. to provide products or services for human beings	GlobeLand30: artificial surface (excluding the range of living space defined by SEDAC)
The living space	3- Urban living space	Including residential land, public management and service land, etc., the living space of urban residents	SEDAC: the population density is greater than 1500/km^2^
	4- Rural living space	Human living space outside the urban built-up area	SEDAC: the population density is 300–1500 /km^2^
The ecological space	5- Forest ecological space	Forests have important ecological functions such as windbreak, sand fixation, and water conservation.	GlobeLand30: forest, bush
	6- Grassland ecological space	Grassland can fix sand and soil and maintain ecosystem balance	GlobeLand30: grass
	7- Water ecological space	The water body stabilizes the regional temperature and has the ability to purify the environment by itself. It is an important ecological land.	GlobeLand30: wetlands, water, glaciers and permanent snow cover
	8- Other ecological spaces	Lands such as sandy land and unused land are difficult to develop and utilize, so their ecological service functions are maintained	GlobeLand30: tundra, bare land

Step 3: Evaluation of data accuracy. On the basis of high-resolution images obtained using Google Earth and the “random sampling” tool in ArcGIS 10.5 software, verification points were randomly selected worldwide. The overall accuracy and Kappa coefficient of the global production–living–ecological space database were calculated, and the confusion matrix was summarized based on PontiusMatrix42.xlsx to assess the accuracy of the data^19^.

### Input data sources

Table 2 shows the data sets utilized for this study. The data resolution of GlobeLand30 is 30 m × 30 m, and the data adopts the WGS-84 coordinate system. The classified images used are mainly 30 m multispectral images, and the data for the 2020 edition employed 16 m high-resolution No. 1 (GF-1) multispectral images. The total number of frames in the GlobeLand30 V2000 edition and the V2010 edition is 853, and the total number of frames in the GlobeLand30 V2020 edition is 966. In terms of data accuracy, 80 maps were extracted from the 853 pieces of global data in GlobeLand30 V2010, and more than 150,000 test samples were laid. The overall accuracy of the GlobeLand30 V2010 data is 83.50%, with a Kappa coefficient of 0.78. On the basis of the landscape shape index sampling model, in the GlobeLand30 V2020 data, a whole set of data points was distributed, resulting in a total of more than 230,000 samples. The overall accuracy of the GlobeLand30 V2020 data is 85.72%, with a Kappa coefficient of 0.82. The GlobeLand30 land cover/land use dataset was used to classify agricultural production space, woodland ecological space, grassland ecological space, water ecological space and other ecological space according to the global production–living–ecological space classification system.

**Table 2:** Summary of the main characteristics of the datasets.

Dataset	Resolution	Data source	Year
GlobeLand cover data	30 m	http://www.globallandcover.com/	2000, 2010, 2020
SEDAC population density data	1 km	https://sedac.ciesin.columbia.edu/	2000, 2010, 2020

The population count grid determined using the SEDAC population density data, which employs the WGS-84 coordinate system with a data resolution of 1 km, consists of population estimates (population per pixel) in 2000, 2005, 2010, 2015 and 2020, which is consistent with national census and population registration data^20^. We took population data from 2000, 2010 and 2020. On the basis of the urbanization classification criteria and population density, the artificial land surface in GlobeLand30 land cover/land use dataset was divided into urban living space, rural living space and industrial production space. SEDAC population density data can be replaced with Landscan global population density spatial distribution data with a spatial resolution of 1 km. The data start time is 2000, and the data are issued annually.

### Product dataset

**Fig. 2:**
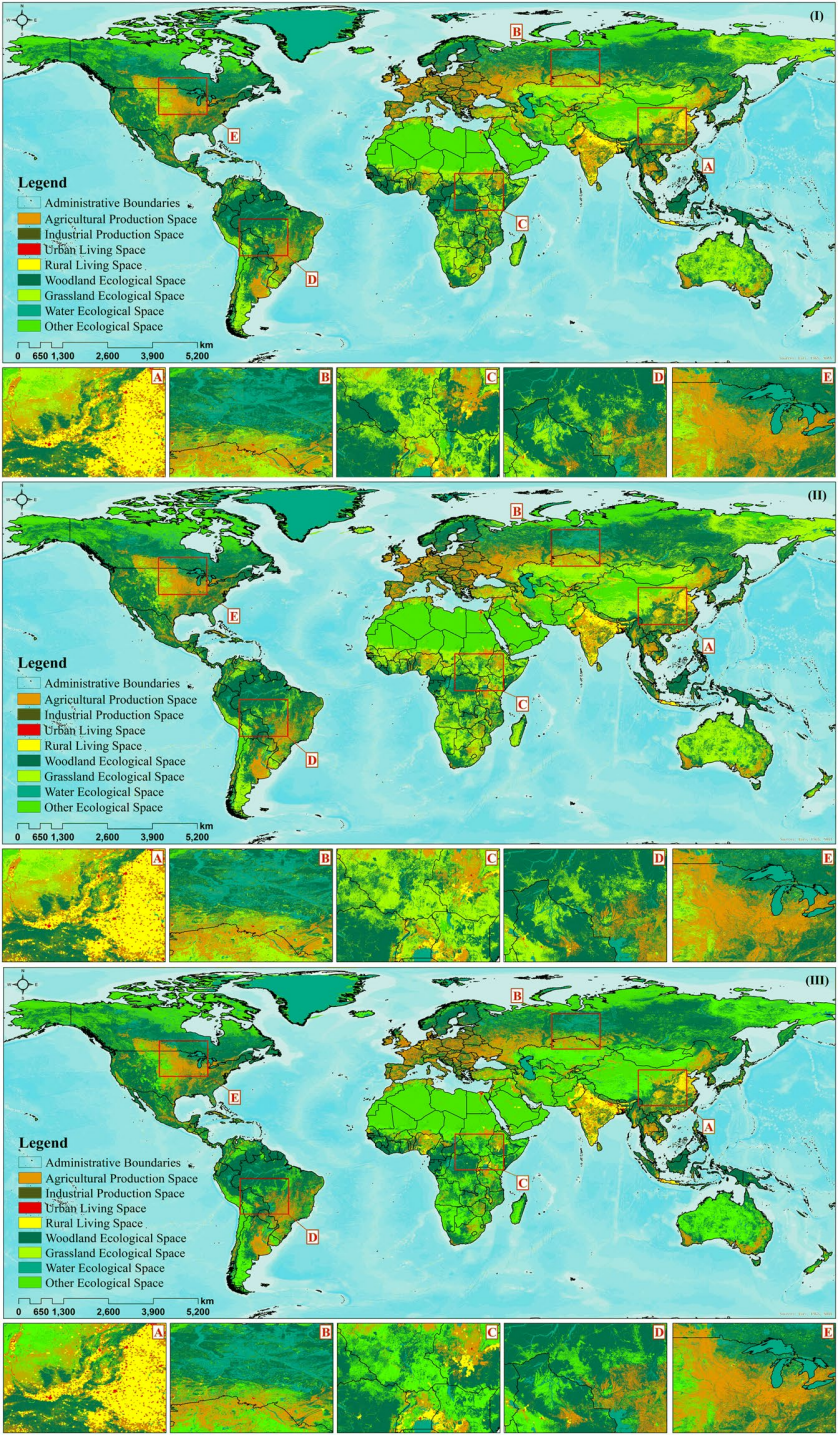
Global production–life–ecological space classification in 2000 (I), 2010 (II), and 2020 (III).

The final product dataset of global production–living–ecological space classification in 2000, 2010 and 2020 is as follows (Fig. 2).

## Data Records

The data is freely available through the Figshare data publisher^21^. The spatial resolution of this dataset is 1 km, and the time step is 10 years. There are three phases from 2000 to 2020. All data are stored in the commonly used geo-tiff format, and the coordinate system is WGS-84, which can be easily obtained using ARCGIS, ENVI, MATLAB and other tools. Within each geo-tiff file, there are different LULC types: 1—Agricultural production space; 2—Industrial production space; 3—Urban living space; 4—Rural living space; 5—Forest ecological space; 6—Grassland ecological space; 7—Water ecological space; and 8—Other ecological spaces. Each geo-tiff file is named “Year”.tif, where “Year” indicates the year of the data.

## Technical Validation

### Layout of verification points

**Fig. 3:**
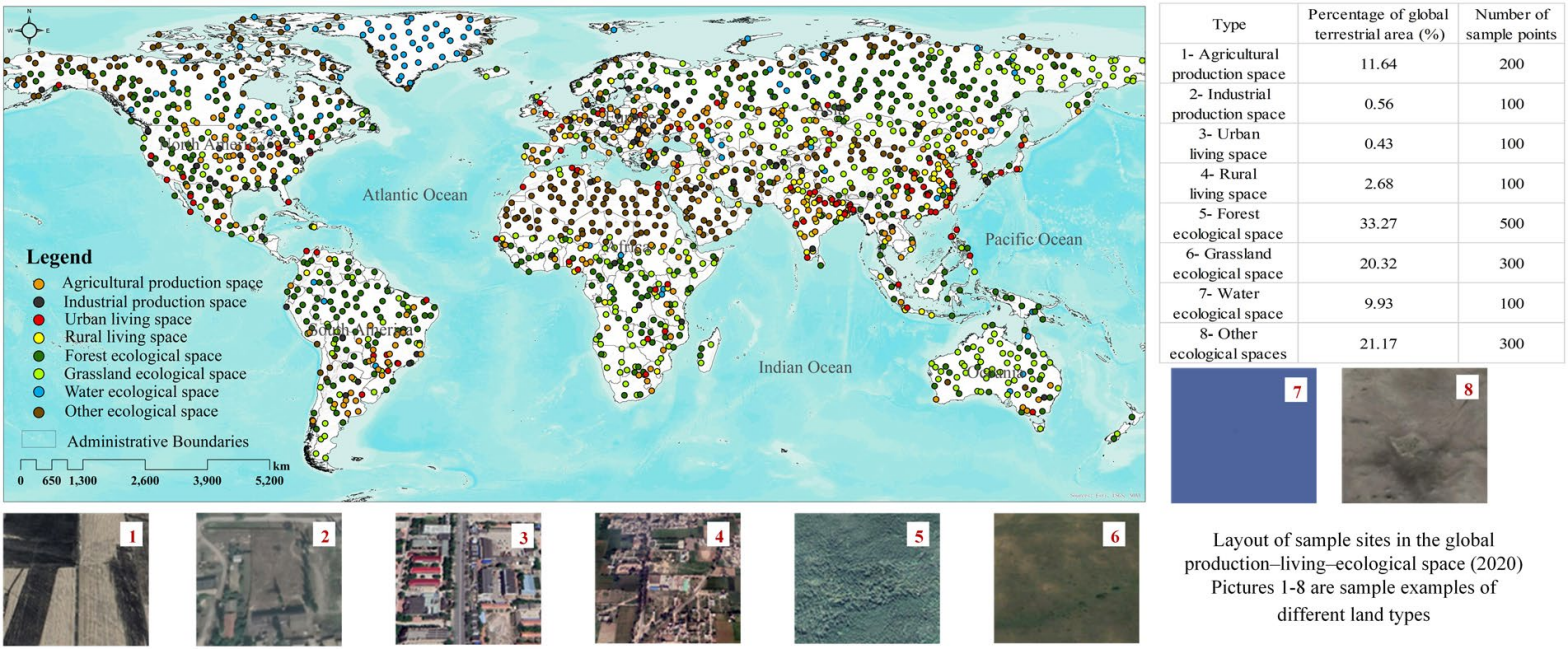
Distribution of sample points in the global production–living–ecological space in 2020. On the right are the layout of sample sites in the global production–living–ecological space (2020).

For large-scale land cover verification, the sample number for land cover type were determined mainly according to the verification cost, area, or the subjective experience of experts, and the sample distribution was usually determined by cluster random sampling, stratified random sampling, or systematic sampling^22–25^. The background data of global production–living–ecological space was the GlobeLand30 land cover/land use dataset. The key operations correspond to the classification system of land type and production–living–ecological space, and subdividing the artificial surface into industrial production space, urban living space and rural living space. The random sampling function of ArcGIS 10.5 is used for verification. However, considering the factors such as the significant differences in the area of different land types in the region, the sample points of spatial types with larger areas may need to be more sparsely sampled in some areas. In comparison, sampling spatial types with smaller areas may need more concentration. At the same time, the higher the number of sample points is, the more reliable the accuracy obtained, so supplementary sampling is performed based on the number of local samples. The verification workflow is the same for each year, so the distribution of sample points in 2020 is taken as an example in Fig. 3.

### Verification point interpretation

The verification point interpretation mainly uses high-resolution images from Google Earth as the reference data source, and the time priority is set as the growing seasons of 2000, 2010 and 2020. The time range can be expanded with the help of relevant knowledge. The interpretation process mainly consists of: establishment of basic interpretation units; comprehensive interpretation according to the shape, shadow, tone and position of samples; and marking all sample points to record relevant information. Since the spatial resolution set for the global production–living–ecological space dataset is 1 km, the basic interpretation unit is a 1000 m × 1000 m square with the sample point as the center, and the dominant land class within the region is taken as the dividing standard.

### Verification results

The accuracy evaluation method for land cover products is mainly used to perform sampling verification of data products, and to obtain the confusion matrix, which is the cross tabulation of classification results and real results (reference data results). The obfuscation matrix is a comparison array obtained by calculating the pixels of the classified dataset and the reference dataset. It is an important method in image accuracy evaluation^23,26^. Overall Accuracy (OA) represents the proportion of correctly classified area in all types. The Kappa coefficient (K) is a comprehensive index used to evaluate the accuracy and consistency of the classification results. The range of the Kappa coefficient is from 0 to 1. The larger the value, the higher the reliability of the data results. However, in some cases the Kappa Index may be useless, misleading or flawed^27–30^. Therefore, we combine the accuracy evaluation and map comparison using two more straightforward parameters proposed by Pontius Jr based on PontiusMatrix42.xlsx (which can be obtained from the index (clarku.edu)): the number difference and the distribution difference^19,31^. The calculation formula for each index is presented below:

1 $$\begin{array}{c}OA=\frac{{\sum }_{i=1}^{r}{n}_{ii}}{N}\end{array}$$

2 $$\begin{array}{c}K=\frac{N{\sum }_{i=1}^{r}{n}_{ii}-{\sum }_{i=1}^{r}{n}_{i* }{n}_{* i}}{{N}^{2}-{\sum }_{i=1}^{r}{n}_{i* }{n}_{* i}}\end{array}$$

where N is the total number of pixels; *n*_*ii*_ is the number of correctly classified pixels; *n*_i*_ is the number of pixels of a certain type in the data to be evaluated; n_*i_ is the number of pixels of a certain type in the reference data; r is the number of classifications. PontiusMatrix42.xlsx has more formulas than can be listed here. For more specific formulas, see (Pontius *et al*. Metrics That Make a Difference: How to Analyze Change and Error. 2022. 10.1007/978-3-030-70765-1).

**Fig. 4:**
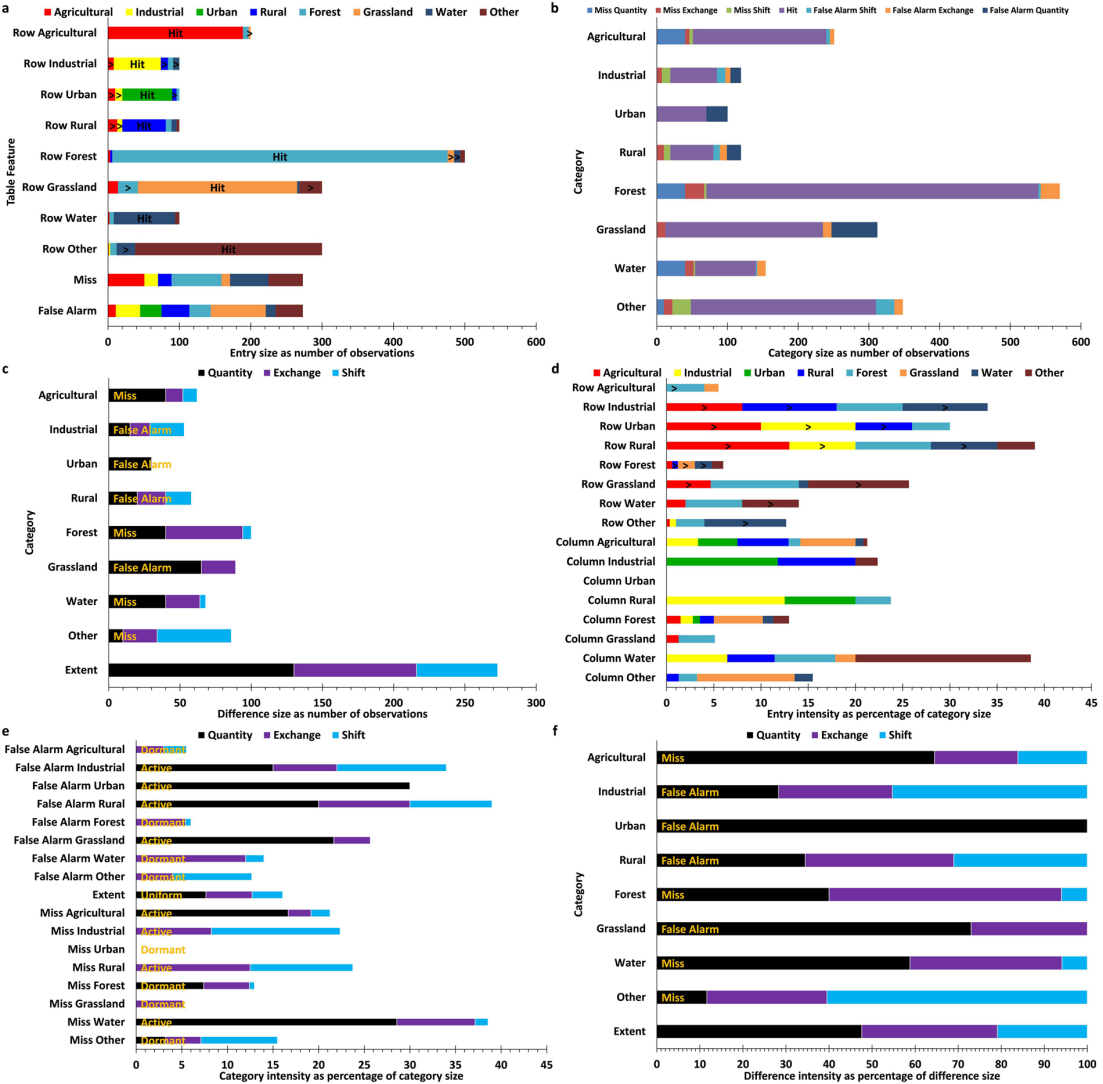
Results in terms of the number of sample sites and the size and intensity of the exchange component in 2020.

The sample dataset is established through sampling, distribution, interpretation, and inspection. Figure 4 shows the number and intensity of hits and misses and false alarms for the 2020 production–living–ecological space data sample points. From the perspective of miss intensity, industrial production space, rural living space, forest ecological space, grassland ecological space and other ecological space all have a low intensity of below 25%. In contrast, agricultural production, urban living, and water ecological space are relatively poor. In terms of false alarms intensity, except for industrial production space and rural living space, all the other categories are generally lower than 30%, indicating that the accuracy rate of the land categories marked in the dataset is high. At the same time, there is an inevitable overestimation in the artificial surface information (Table 3). The overall accuracy of the global production–living–ecological space data in 2020 was 83.94% and the Kappa coefficient was 0.81. The overall accuracy of the global production–living–ecological space data in 2010 was 87.00% and the Kappa coefficient was 0.84. The overall accuracy of the spatial data in 2000 was 86.06% and the Kappa coefficient was 0.83.

**Table 3:** Miss intensity and False alarm intensity of various surface types from 2000 to 2020.

Class	2000		2010		2020	
	Miss intensity (%)	False alarm intensity (%)	Miss intensity (%)	False alarm intensity (%)	Miss intensity (%)	False alarm intensity (%)
Agricultural production space	25.53	12.5	21.49	10.5	21.25	5.5
Industrial production space	2.80	29.00	19.76	35.00	22.35	34.00
Urban living space	25.90	20.00	0.00	10.00	0.00	30.00
Rural living space	18.99	36.00	16.10	27.00	25.00	39.00
Forest ecological space	5.2	8.00	10.14	7.80	11.65	6.00
Grassland ecological space	17.06	17.33	7.70	16.00	5.20	25.67
Water ecological space	25.83	11.00	25.66	16.00	38.57	14.00
Other ecological Spaces	7.69	8.00	8.95	8.33	15.48	12.67

On the whole, the land cover information products of global production–living–ecological space data reflect the distribution of global surface cover. They provide useful data for studying the impact of human activities on the Earth’s surface. However, due to the limitation of data resolution, a finer resolution cannot be reached, which may lead to less accurate boundary delineation in spatial types; secondly, the delineation of industrial production space and living space based on the artificial surface is also a fundamental limitation, which is limited by the difference in the accuracy of population data and delineation standards, which may affect our ability to make fine distinctions between industrial production space as well as living space. We will consider using a more detailed approach to segment the production space in the future. Suppose the data type and resolution of nighttime lighting data and socioeconomic data such as GDP are suitable (socioeconomic data are generally counted in administrative units). In that case, we will consider using more data to identify the production space. This will improve our understanding and accuracy of the production space and will also help improve the quality of our research.

## Usage Notes

The global production–living–ecological space dataset provides a reference dataset formed of superimposed global land cover and land use data and population density data. In this sense, it provides a unique dataset for studying global land cover and land use. This dataset has many potential uses, including climate change and carbon emission studies, urbanization studies, and agriculture and food security studies. By analyzing changes in production, living and ecological space, the impact of different land use types on carbon cycling and storage can be assessed. Thus the potential for carbon emissions and sequestration can be estimated, thus supporting the development of policies and measures to mitigate climate change^32,33^. In addition, this dataset can be used to study urbanization processes and patterns of urban expansion and to understand the effects of urbanization on land use types and spatial distribution, as well as environmental, social, and economic impacts^34,35^. For agriculture and food security research, this dataset can help analyze the distribution and utilization of agricultural land, assess land arable and agricultural capacity, study the relationship between agricultural production and food security, and support agricultural development strategies and policy formulation. These potential uses are very interesting and meaningful to readers. By publishing these data, others will find new ways and means to optimize the use of land cover, land use data and socio-economic statistics. Due to the qualitative limitations of these data, they cannot be considered validation data, so we refer to them as reference data.

## Data Availability

No custom code has been used during the generation and processing of this dataset.

## References

[1] Long HL, Liu YQ, Li TT, Wan J (2014). Spatial interlinking of land use planning and environmental protection planning from the perspective of ecological civilization construction. Econ. Geogr.

[2] Fei JB (2019). Research progress of ecological space and ecological land in China. Chin. J. Eco-Agric.

[3] Long HL, Liu YQ, Li TT, Wan J, Liu AC (2015). A primary study on ecological land use classification. Ecology and Environmental Sciences.

[4] Yu F (2015). Study of ecological land in China: conception, classification, and spatial-temporal pattern. Acta Ecol. Sin..

[5] Robert C (2014). Changes in the global value of ecosystem services. Glob Environ Change.

[6] Deng HB, Chen CD, Liu X, Wu G (2009). Conception and function classification of regional ecological land. Acta Ecol. Sin..

[7] Xu J, Zhou YK, Jin XB, Yi LQ (2007). Discussing virgin land vlassification subsystem based on the protection of the eco-environment. Resources Science.

[8] Liu J, Liu Y, Li Y (2017). Classification evaluation and spatial-temporal analysis of “production-living-ecological” spaces in China. Acta Geogr Sin.

[9] Li K, Zhang BY, Xiao WD, Lu Y (2022). Land use transformation based on production−living−ecological space and associated eco-environment effects: a case study in the Yangtze River Delta Urban Agglomeration. Land.

[10] Zhao L, Zhang GJ, Zhu YM, Zhang PT, Xu HU (2017). Land multi-functional transformation and characteristic analysis based on land use transition: a case study of Tang county, Hebei province. China Land Sci.

[11] Zhang HQ, Xu EQ, Zhu HY (2017). Ecological-living-productive land classification system in China. Journal of Resources and Ecology.

[12] Liu C, Xu XQ, Sun PL, Liu J (2016). Progress and prospects of multi-functionality of land use research. Prog Phys Geogr.

[13] Zhang H, Xu E, Zhu H (2015). China’s production–living–ecological space classification and its space inter-pattern. Resource Science.

[14] United Nations. *Sustainable Development Goals.* https://sdgs.un.org/ (2015).

[15] Lowe I (2001). Sustainability science. Science.

[16] Dijkstra L, Florczyk AJ, Freire S (2021). Applying the Degree of Urbanisation to the globe: A new harmonised definition reveals a different picture of global urbanisation. J Urban Econ.

[17] Melchiorri M, Pesaresi M, Florczyk AJ (2019). Principles and Applications of the Global Human Settlement Layer as Baseline for the Land Use Efficiency Indicator—SDG 11.3.1. ISPRS Int. J. Geo-Inf..

[18] (2021).

[19] Pontius, R. G. *Metrics that make a difference: how to analyze change and error* (Springer Nature: Cham, Switzerland, 2022).

[20] (2018). NASA Socioeconomic Data and Applications Center (SEDAC).

[21] Fu JY, Gao Q, Jiang D, Li X, Lin G (2023). figshare.

[22] Wang Y, Zhang JX, Di L, Yang WJ, Zhang WL (2018). Accuracy assessment of GlobeLand30 2010 land cover over China based on geographically and categorically stratified validation sample data. Remote Sens..

[23] Wang ZX, Liu C, Tun WN (2019). Google earth images based land cover data validation dataset for globeland30 (2010) in the region of roof of the world. Journal of Global Change Data & Discovery.

[24] Foody GM (2002). Status of land cover classification accuracy assessment. Remote Sens Environ.

[25] Stehman SV, Olofsson P, Woodcock CE, Herold M, Friedl MA (2012). A global land-cover validation data set, II: augmenting a stratified sampling design to estimate accuracy by region and land-cover class. Int J Remote Sens.

[26] Congalton RG (1991). A review of assessing the accuracy of classifications of remotely sensed data. Remote Sens Environ.

[27] Foody GM (2020). Explaining the unsuitability of the kappa coefficient in the assessment and comparison of the accuracy of thematic maps obtained by image classification. Remote Sens Environ.

[28] Pontius RG, Millones M (2011). Death to kappa: birth of quantity disagreement and allocation disagreement for accuracy assessment. Int J Remote Sens.

[29] Pontius RG, Parmentier B (2014). Recommendations for using the relative operating characteristic (ROC). Landscape Ecol..

[30] Pontius RG, Si K (2014). The total operating characteristic to measure diagnostic ability for multiple thresholds. International Journal of Geographical Information Science.

[31] Varga OG, Pontius RG, Szabó S (2019). Intensity Analysis and the Figure of Merit’s components for assessment of a Cellular Automata – Markov simulation model. Ecol. Indic..

[32] Wei GE, Bi M, Liu X, Zhang ZK, He BJ (2023). Investigating the impact of multi-dimensional urbanization and FDI on carbon emissions in the belt and road initiative region: Direct and spillover effects. J. Clean. Prod..

[33] Verburg PH, Neumann K, Nol L (2011). Challenges in using land use and land cover data for global change studies. Glob Chang Biol.

[34] Chen GZ (2020). Global projections of future urban land expansion under shared socioeconomic pathways. Nat Commun.

[35] Wei GE (2023). Evolutionary trends of urban expansion and its sustainable development: Evidence from 80 representative cities in the belt and road initiative region. Cities.

